# Supplementary material for: An investigation of the pigments, antioxidants and free radical scavenging potential of twenty medicinal weeds found in the southern part of Bangladesh
Source: PeerJ. 2024 Jul 23;12:e17698. doi: 10.7717/peerj.17698 (PMC11276756; doi:10.7717/peerj.17698)
Supplement: Supplemental Information 1 [file peerj-12-17698-s001.docx]

**Supplementary Table S1:** List of 20 medicinal weeds species used in the study for screening of pigments, phytochemical properties and free radical scavenging potential

| **Scientific Name** | **Common/English name** | **Family** | **Pictures** |
| --- | --- | --- | --- |
| *Acalypha indica* L. | Indian nettle | [Euphorbiaceae](https://www.sciencedirect.com/topics/pharmacology-toxicology-and-pharmaceutical-science/euphorbiaceae) | 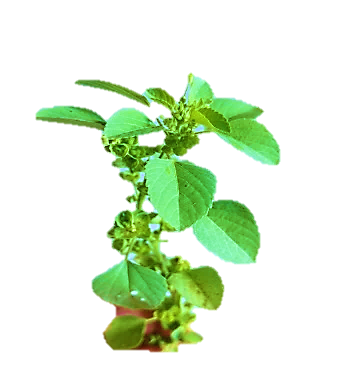 |
| *Ageratum conyzoides L.* | Billygoat Weed | Asteraceae | 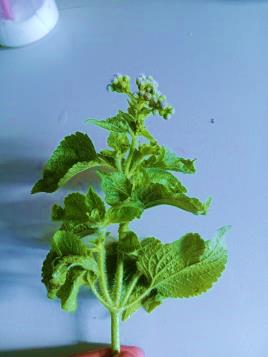 |
| *Alternanthera sessilis* L. | Sessile joyweed | Amaranthacea | 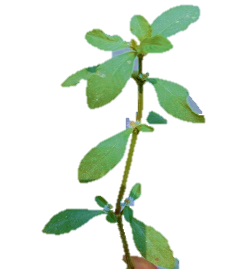 |
| *Bryophyllum calycinum* S. | Mother of thousands | Crassulaceae | 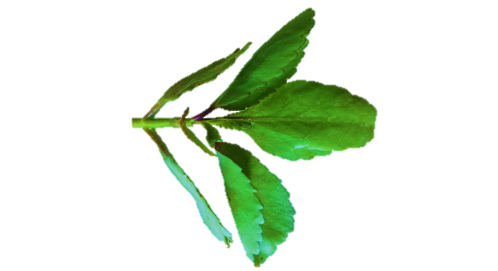 |
| *Centella verticillata* T*.* | Whorled pennywort | *Araliaceae* | *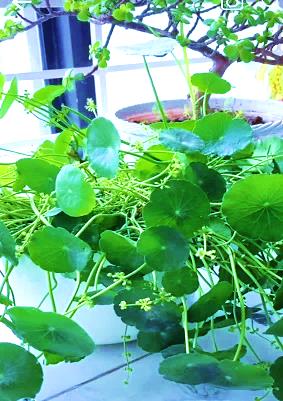* |
| *Coccinia grandis* L*.* | Ivy gourd | *Cucurbitaceae* | *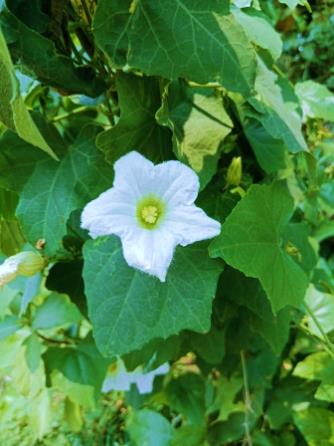* |
| *Eclipta postrata* L. | False daisy | *Asteraceae* | *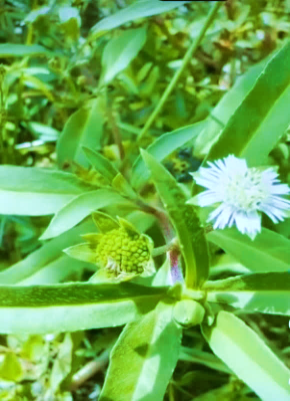* |
| *Enhydra fluctuans* Lour. | Water spinach | *Asteraceae* | *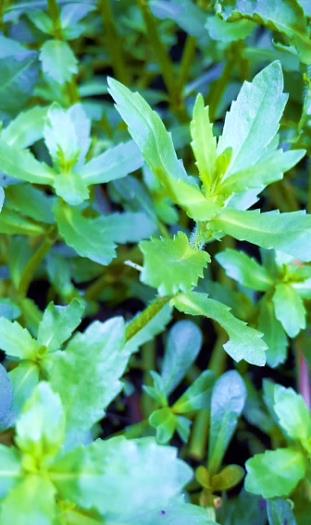* |
| *Euphorbia hirta*  L. | Asthma plant | [Euphorbiaceae](https://www.sciencedirect.com/topics/pharmacology-toxicology-and-pharmaceutical-science/euphorbiaceae) | 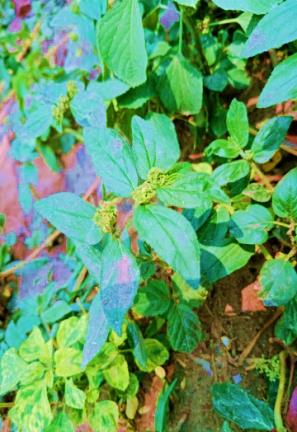 |
| *Heliotropium indicum* L. | Indian turnsole | *Boraginaceae* | *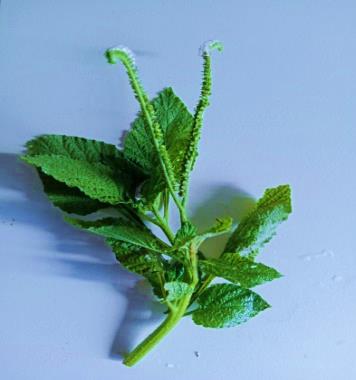* |
| *Oxalis corniculata* L*.* | Creeping wood sorrel | *Oxalidaceae* | *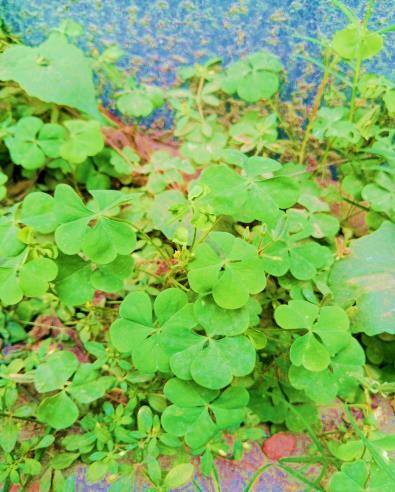* |
| *Parthenium hysterophorus* L. | Famine weed | Asteraceae | 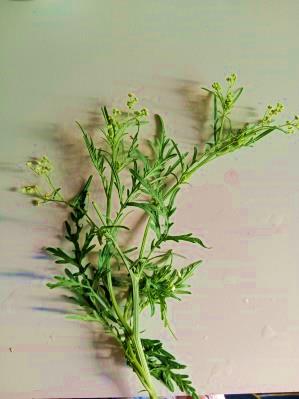 |
| *Persicaria lapathifolia* L. | Pale smartweed L. | Polygonaceae | 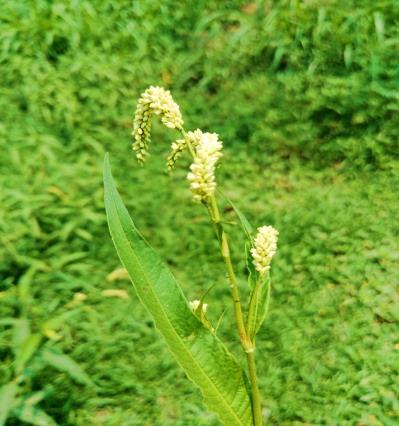 |
| *Portulaca oleracea* L. | Common purslane | Portulacaceae | 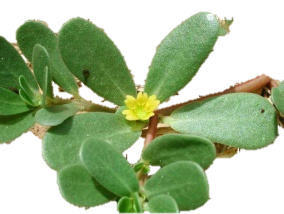 |
| *Ruellia tuberosa* L. | Minnieroot | Acanthaceae | 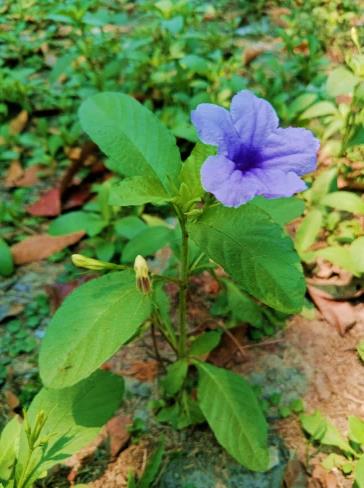 |
| *Scoparia dulcis* L. | Sweet broom weed | Scrophulariaceae | 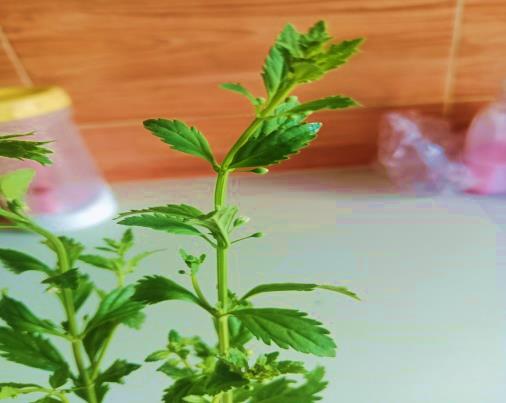 |
| *Senna occidentalis* L. | Coffee senna | Fabaceae | 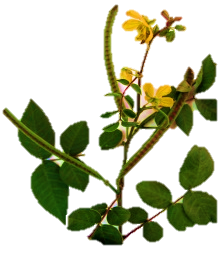 |
| *Synedrella nodiflora* L. | Node weed | Asteraceae | 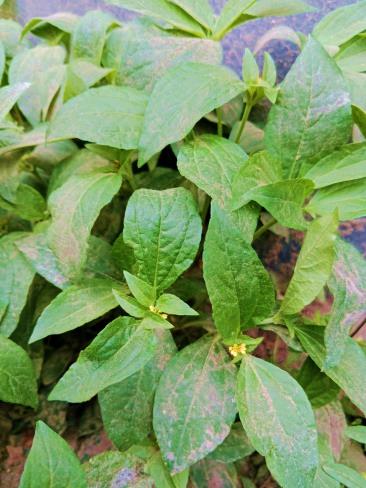 |
| *Trema orientalis* L. | Charcoal tree | Cannabaceae | 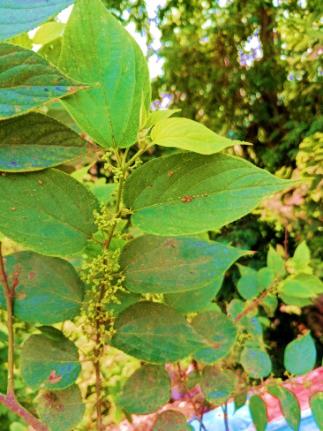 |
| *Tridax procumbens* L. | Coat buttons | Asteraceae | 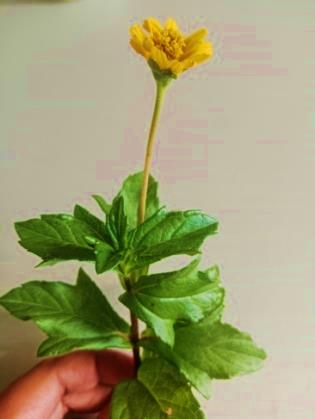 |
